# Computational anatomy and geometric shape analysis enables analysis of complex craniofacial phenotypes in zebrafish

**DOI:** 10.1242/bio.058948

**Published:** 2022-02-17

**Authors:** Kelly M. Diamond, Sara M. Rolfe, Ronald Y. Kwon, A. Murat Maga

**Affiliations:** 1 Center for Developmental Biology and Regenerative Medicine, Seattle Children's Research Institute, Seattle, WA 98101, USA; 2 Friday Harbor Marine Laboratories, University of Washington, San Juan, WA 98250, USA; 3 Department of Orthopedics and Sports Medicine, University of Washington, Seattle, WA 98195, USA; 4 Institute for Stem Cell and Regenerative Medicine, University of Washington, Seattle, WA 98109, USA; 5 Division of Craniofacial Medicine, Department of Pediatrics, University of Washington, Seattle, WA 98105, USA

**Keywords:** Cranial morphology, Osteogenesis imperfecta, Geometric morphometrics, Computational anatomy

## Abstract

Due to the complexity of fish skulls, previous attempts to classify craniofacial phenotypes have relied on qualitative features or sparce 2D landmarks. In this work we aim to identify previously unknown 3D craniofacial phenotypes with a semiautomated pipeline in adult zebrafish mutants. We first estimate a synthetic ‘normative’ zebrafish template using MicroCT scans from a sample pool of wild-type animals using the Advanced Normalization Tools (ANTs). We apply a computational anatomy (CA) approach to quantify the phenotype of zebrafish with disruptions in *bmp1a*, a gene implicated in later skeletal development and whose human ortholog when disrupted is associated with Osteogenesis Imperfecta. Compared to controls, the *bmp1a* fish have larger otoliths, larger normalized centroid sizes, and exhibit shape differences concentrated around the operculum, anterior frontal, and posterior parietal bones. Moreover, *bmp1a* fish differ in the degree of asymmetry. Our CA approach offers a potential pipeline for high-throughput screening of complex fish craniofacial shape to discover novel phenotypes for which traditional landmarks are too sparce to detect. The current pipeline successfully identifies areas of variation in zebrafish mutants, which are an important model system for testing genome to phenome relationships in the study of development, evolution, and human diseases.

This article has an associated First Person interview with the first author of the paper.

## Abstract (precis)

**Summary:** A computational anatomy approach offers a potential pipeline for high-throughput screening of complex zebrafish craniofacial phenotypes, an important model system for the study of development, evolution, and human diseases.

## INTRODUCTION

The fish craniofacial skeleton is a useful system for elucidating genetic and environmental contribution to phenotype in vertebrates. In the context of development, studies have focused on the genetic mechanisms that shape the cranial skeleton (Kimmel et al., 2020; Miller et al., 2007). Craniofacial analyses have been used to understand the pathways that have enabled morphological evolution (Kimmel et al., 2005), phenotypic plasticity (Navon et al., 2020), and adaptive radiations (Powder and Albertson, 2016) in fishes. Additionally, zebrafish are developing as a model system for quantifying phenotypic variability associated with human bone diseases, such as Osteogenesis Imperfecta (Busse et al., 2019; Gistelinck et al., 2018; Kwon et al., 2019). A longstanding challenge to analyzing the fish craniofacial skeleton is accurately capturing phenotypes that involve subtle alterations and complex 3D changes, including potential asymmetric alterations.

The traditional methods for quantifying cranial morphology use manually-placed homologous landmark points on 2D images of the lateral view of the head (i.e. Sidlauskas, 2008). However, manual placement limits potential for rapid-throughput applications. Further, the requirement for homologous structures limits landmark placement across the skull, and hence may miss the phenotypic variation in these areas. While MicroCT can help realize 3D structures, 3D landmark placement is complex as visualizations are dependent on both the scanner and rendering software settings used. Moreover, because of the close proximity of bones, segmentation-based approaches that are useful for axial skeleton are not amenable to those in the head. There is an urgent need to develop robust methods for phenotyping in the craniofacial skeleton that are sensitive to complex 3D changes while being amenable to rapid-throughput analyses.

Here, we propose using an atlas-based computational anatomy (CA) approach to build a reference template of the zebrafish skull and then using a pseudo-landmark pipeline to identify areas of the skull that vary among mutant and wild-type fish. Atlas-based approaches estimate an unbiased anatomical ‘template’ from a group of images (Guimond et al., 2006) and then use this template as the basis to assess shape differences among groups of interest (Ashburner and Friston, 2000). Atlas-based approaches have been used to characterize phenotypes in many neuroimaging studies in humans and fetal mice (Mandal et al., 2012; Mazziotta et al., 2001; KOMP2 project), as well as in the cranial skeleton of humans and mice (Darvann et al., 2011; Maga et al., 2017; Toussaint et al., 2021). We define pseudo-landmarks here as landmarks that are not morphologically homologous, but instead, are placed automatically on the surface of the template and transferred to each specimen, enforcing a degree of geometric homology.

We apply these methods to zebrafish with mutations in *bmp1a*, a gene implicated in later skeletal development. In humans, Bone Morphogenetic Protein 1 (BMP1) encodes for a secreted protein involved in procollagen processing. Individuals with mutations in BMP1 exhibit increased bone mineral density and recurrent fractures characteristic of Osteogenesis Imperfecta (OI; Asharani et al., 2012). Severe forms of OI are frequently associated with craniofacial abnormalities (Dagdeviren et al., 2019). Previous work in *bmp1a* and other zebrafish OI models have identified phenotypic abnormalities in the axial skeleton (Hur et al., 2017). However, due to the complicated structure of the fish cranial skeleton, craniofacial abnormalities in zebrafish OI models have mostly focused on qualitative phenotypes (Gistelinck et al., 2018), and little work has been done to quantify complex cranial phenotype. Here, we report complex craniofacial phenotype arising from disruptions in *bmp1a*. Our methods aim to identify areas of greatest variation among mutant and wild-type fish cranial phenotypes with minimal user intervention. We envision this method as a high-throughput first pass to identify areas for further exploration for phenotype-genotype associations in the skeletal system.

## RESULTS AND DISCUSSION

When analyzing otoliths, we did not find significant differences between manually segmented volumes and atlas segmented volumes (t=-0.912, *P*=0.363; Fig. S1), though there were differences between some of the individual otoliths (Table S1, Fig. S1). The most apparent difference between *bmp1a* and wild-type fish is that *bmp1a* fish have larger otoliths than wild-type fish, especially for the asteriscus, the largest otoliths in the zebrafish. This difference was consistent in both manually and CA segmented otoliths (Table 1; Fig. S1). In contrast to bone formation, in which the mineral phase is primarily hydroxyapatite, otoliths are formed via an accumulation of calcium carbonate in the acellular endolymph of the fish inner ear (Payan et al., 2004). Previous work found higher tissue mineral density in *bmp1a* fish across the axial skeleton (Hur et al., 2017) and this result suggests potential influence of *bmp1a* on other pathways associated with mineralized tissues.

**Table 1.**
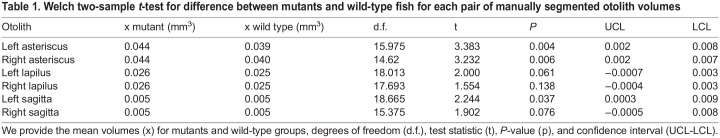
Welch two-sample *t*-test for difference between mutants and wild-type fish for each pair of manually segmented otolith volumes

**Fig. 1.**
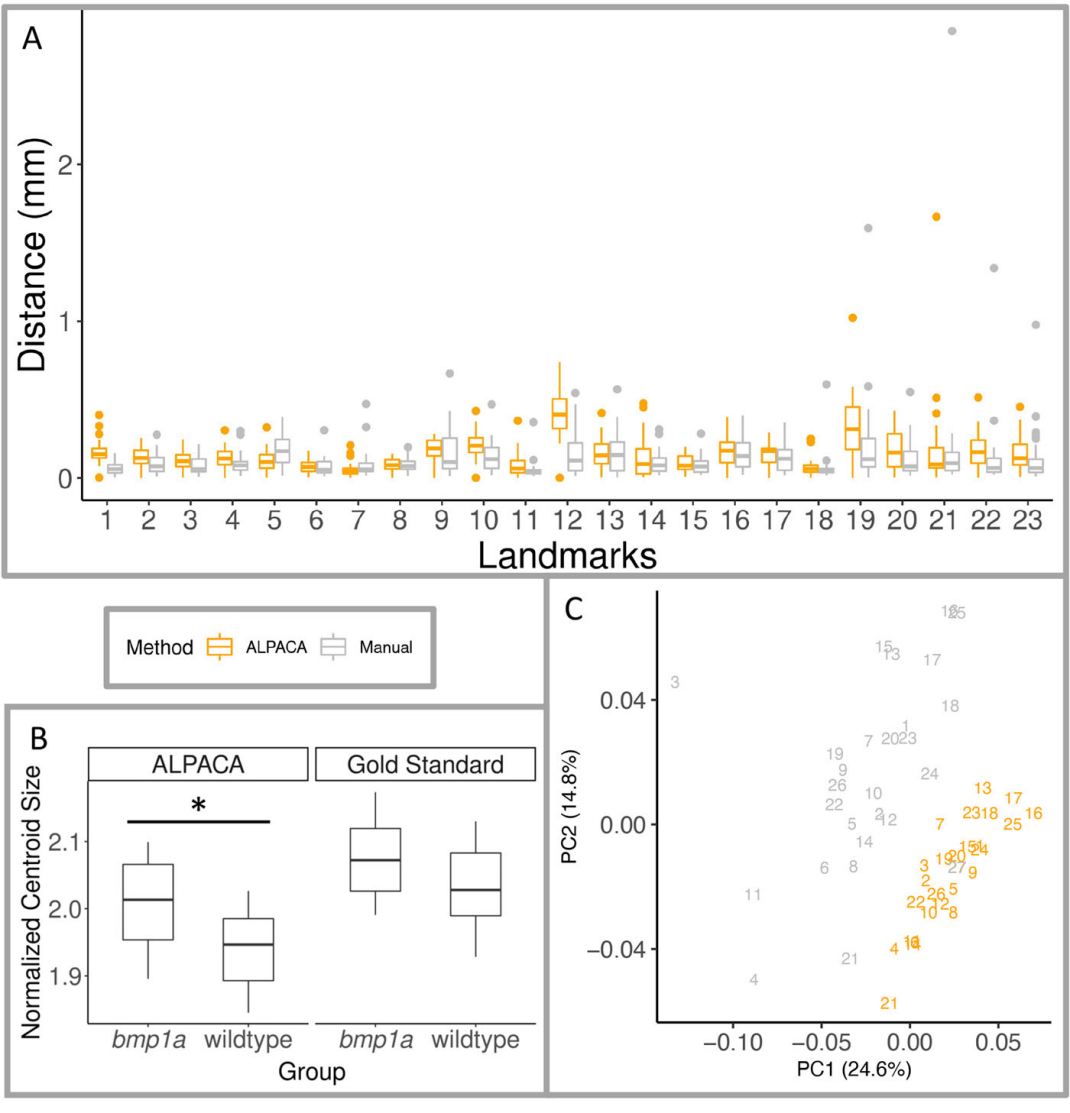
**Pipeline validation using 23 traditional landmarks.** Boxplots are shown for (A) Euclidean distance between Gold Standard landmark locations and ALPACA transferred points (orange), and distance between the two manual landmark placements (grey), where color indicates method used for all panels, and (B) normalized centroid size of gold standard and ALPACA transferred pseudo-landmarks. Midline of boxplots show median value, with hinges corresponding to first and third quartiles, and whiskers extending to largest and smallest value no further than 1.5 times the interquartile range. Also shown (C) are the first two principal components of shape space from the combined GPA analysis of gold standard and ALPACA transferred 23 landmarks. The percent of variance for each PC is show in parenthesis of each axis. Individual fish are depicted as different numbers, fish 1–12 are crispant fish, fish 13–26 are wild-type fish, and fish 27 is the atlas.

We found a similar pattern in normalized centroid size, with the pseudo-landmark (t=2.700, *P*=0.013) and the trends for the gold standard (t=1.956, *P*=0.063) methods finding *bmp1a* fish to be larger than wild-type fish (Fig. 1B). Previous work on the axial skeleton found that *bmp1a* fish had reduced standard length, but thicker bones (Watson et al., 2020). It is possible that the increase in bone thickness is contributing to the larger normalized centroid size observed in these analyses. Additionally, while these two methods to differ in normalized centroid size (*F*=18.66, *P*<0.001; Fig. 1B), we find similar patterns and across methods, which helps to validate our pseudo-landmark pipeline. The distances between the gold standard and the ALPACA transferred points of the gold standard, as well as the distance between the two independent manual landmark positions. We found that while the manual landmark placement showed shorter distances than the ALPACA method (*F*=14.690, *P*<0.001; Table 2, Fig. 1A), both methods show similar patterns in variation across the landmark points. For further validation of our pipeline, we tested if landmarking method varied across the combined shape space. While we do find differences among manual and ALPACA methods (*F*=9.860, Z=4.883, p=0.001), both methods show similar patterns of variation within the different treatment groups (Fig. 1C).

**Table 2.**
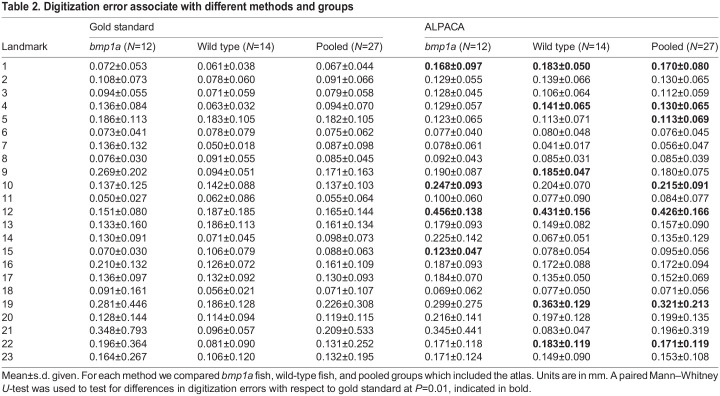
Digitization error associate with different methods and groups

**Fig. 2.**
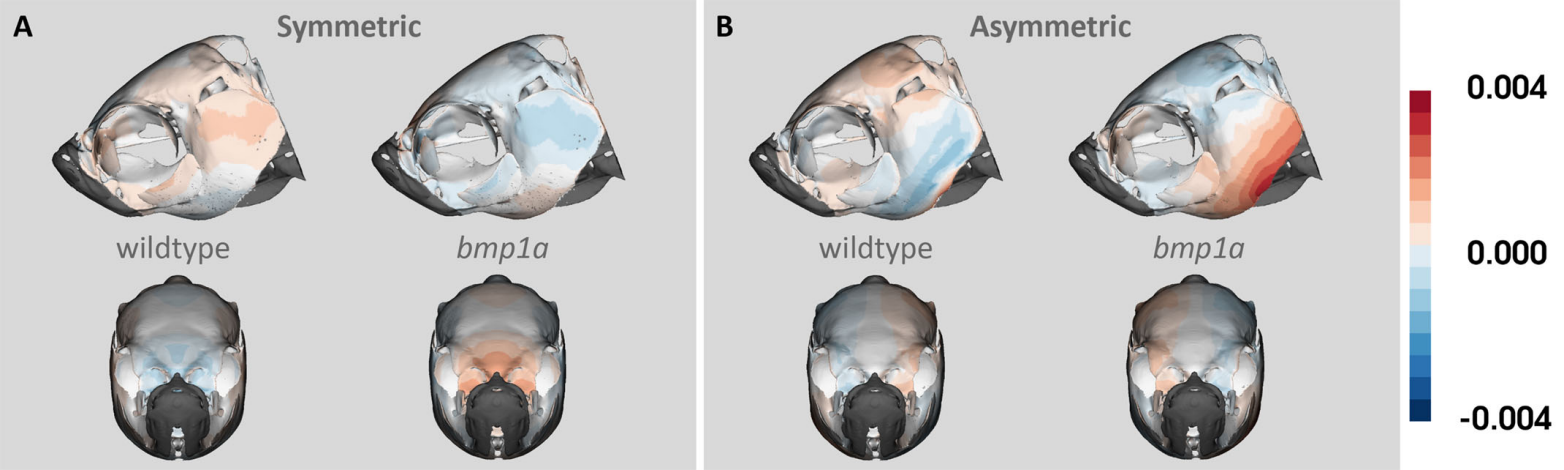
**Heat map of (A) symmetric and (B) asymmetric components of shape variation.** Lateral and anterior views are shown for each group (wild type and *bmp1a*) within both components of shape variation. Colors show variation in shape from the symmetric atlas, with deeper colors representing greater variation from the atlas.

To assess if there were areas of the skull that had local shape variability more subtle than what could be determined from our sparse landmark analysis or manual LM-based Procrustes analysis, we deployed a pseudo-landmark approach, placing 372 geometrically placed pseudo-landmarks across the outer surface of the cranial skeleton. In our symmetry analysis of pseudo-landmark points, we found significant differences in symmetry between groups for both the symmetric (*F*=3.573, Z=2.708, *P*=0.011) and asymmetric (*F*=3.830, Z=3.124, *P*=0.002) components of shape variation. The symmetric differences in shape variation between groups were concentrated in the anterior frontal bone and the dorsal portion of the operculum (Fig. 2). While the asymmetric differences between groups were concentrated in the posterior portion of the parietal bone and ventral portion of the operculum (Fig. 2). Tables of PC scores for both analyses can be found in the supplemental information (Tables S2, S3).

**Fig. 3.**
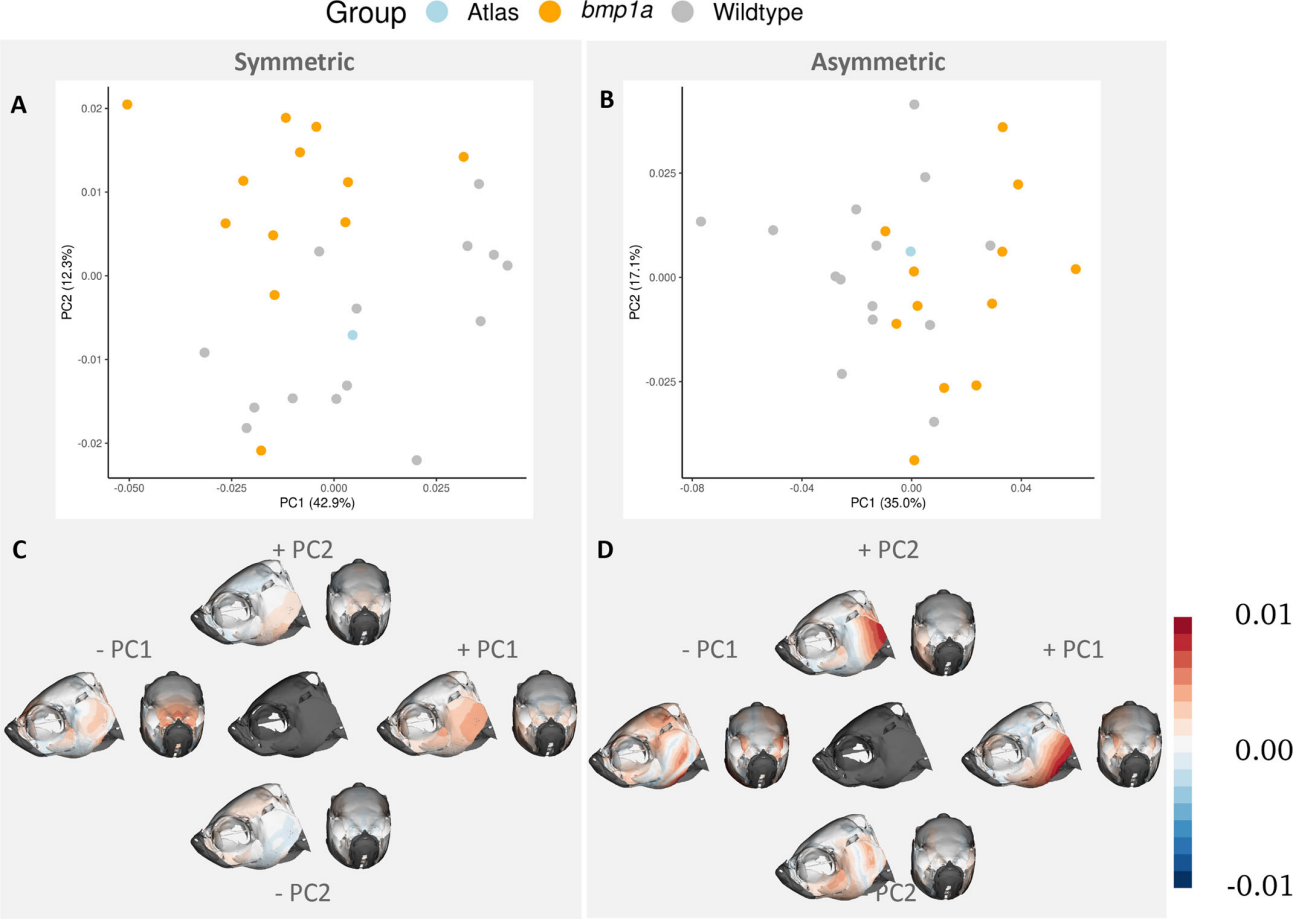
**First two principal components of symmetry analysis.** PC plots show separation of groups (represented by color) along the first and second PCs (A,B). Heat maps of the same PCs represent where shape variation occurs across each axis (C,D). Columns represent symmetric (A,C) and asymmetric (B,D) components of shape variation. The central image in C and D represents mean shape of each component. Color in C and D represents the Procrustes distance between the average shape and the shape occupying the ends of each PC axis. Deeper colors represent larger differences, and the specific colors refer to differences in direction relative to the average image.

The results of separate PCA of each shape component suggest the asymmetric component of shape may be contributing more to the variation between groups in our dataset. For the symmetric component of variation, we found significant differences between *bmp1a* and wild-type fish along PC2, which explained 12.3% of the variation in the data (*F*=7.018; Z=2.006; *P*=0.002), but not along PC1, which explained 42.9% of the variation in the data (*F*=2.583; Z=1.124; *P*=0.092; Fig. 3) or any other PCs. Whereas in the asymmetric shape space, we found differences between groups along PC1, which explained 35.0% of the variation, (*F*=6.305, Z=1.753, *P*= 0.009), but not along PC2, which explained 17.1% of the variation (*F*= 0.318, Z=-0.374, *P*=0.677; Fig. 3), or any other PCs. We find that the greatest variation across the skull is observed in the posterior operculum, as observed along the first PC in both the symmetrical and asymmetrical analyses (Fig. 3). In the symmetric shape analysis, we also observe variation in the anterior portion of the frontal bone across PC1 (Fig. 3). The asymmetric component of PC1 shows variation in the lateral parietal and supraocular regions (Fig. 3). Relative to the asymmetric component, smaller changes (lighter coloration in Fig. 3) are observed in the symmetric component of PC2 in the frontal, parietal, and ventral opercular bones. The asymmetric component of the PC2 axis is again concentrated around the opercular and ocular regions (Fig. 3). As we removed pseudo-landmark points associated with areas of the skull that varied due to preservation or scanning methods, this variation represents areas of interest for exploring how phenotype differs between mutant and wild-type fishes.

As fish skulls are particularly kinetic, it is possible that the differences observed, especially those observed with the opercula, could be the result of preservation and or imaging techniques. We also note that because we used a symmetric template to generate the pseudo-landmark points, fine scale aspects of anatomical variation, such as differences in lateral line pits, may not be captured by our current pipeline. However, we note that this pseudo-landmark method is intended to identify areas of phenotypic variability across complex morphology for which traditional landmarking methods may be too sparse to capture, and not to directly correlate genotype to phenotype. Together these results provide support for phenotypic effects of the *bmp1a* mutation on the cranial phenotype of zebrafish. Future work should expand the number of families to ensure this is not unique to this particular family and include other potential factors, such as differences in sex. We have shown how our pipeline can identify areas of greatest variation among groups of animals. In combination with additional morphological analyses, we hope this pipeline will enable researchers to better define the links between genotype and phenotype.

## MATERIALS AND METHODS

Generation of mutant zebrafish (*Danio rerio*) and collection of MicroCT scans were performed as part of a previous study (Watson et al., 2020). Fish were scanned using a vivaCT40 MicroCT scanner (Scanco Medical, Switzerland), with 21 mm isotropic voxel resolution, 55kVp, 145 mA, 1024 samples, 500proj/180°, 200 ms integration time (Watson et al., 2020). We used a total of 23 wild-type fish from two clutches (‘wildtype fish’) to build our atlas and used 12 *bmp1a* somatic mutants (‘*bmp1a* fish’), from a single clutch. To eliminate clutch-effect, we included only wild-type fish (*N*=14) from the same clutch as the *bmp1a* mutants used in this study to analyze shape differences between groups, as such the final number of samples in the analysis was 26, not including the atlas. Watson et al. (2020) performed a comparison of *bmp1a* somatic and germline mutants and showed that somatic *bmp1a* mutants recapitulate germline *bmp1a* mutant phenotypes but possess additional phenotypic variability due to mosaicism. We focused our analyses on *bmp1a* somatic mutants as they provide a real-world sample of phenotypic variability likely to be encountered in CRISPR-based reverse genetic screens (Shah et al., 2015; Watson et al., 2020).

### Atlas building

To investigate potential asymmetric patterns, we built a symmetrical atlas of wildtype fish (*N*=23) by first reflecting all MicroCT volumes along the sagittal plane using the reflectImage function of the AntsR package in R (Avants, 2020). For each of the 23 fish scanned, two volumes (original and reflected) were used to build the atlas to minimize asymmetries between right and left sides of the atlas. A fully symmetrized atlas was generated using the antsMultivariateTemplateConstruction2.sh script as provided by the Advanced Normalization Tools (ANTs). For a full explanation of adjustable parameters see Avants et al. (2009). The following settings were used to generate the atlas: type of transformation: greedy symmetric normalization (SyN); similarity metric: cross correlation (CC); iterations: 4; N4 bias field correction: off; smoothing factor: 3×2×1; shrinkage factor: 6×4×2. This atlas building script estimates the best average shape by iteratively estimating an average, or template, and computing deformation fields that map each image to the template. The resultant deformation fields are applied to samples, and a new average is estimated and then used as a new reference for the next step of registrations. Four iterations were sufficient to obtain a symmetrical and anatomically detailed template from which individual bones and landmark locations could be identified.

### Atlas validation

To validate our atlas, we compared automated and manual segmentations and landmarks. Before continuing we would like to note the underlying assumption of these comparisons, that the manual dataset represents a ground truth, is likely incorrect (Robinson and Terhune, 2017), and all analyses should be considered in this context.

To quantitatively validate the atlas and our computational anatomy (CA) framework, we first created manual segmentations of individual otoliths from every sample using the open-source 3D Slicer application (Fedorov et al., 2012). We chose otoliths because they are dense, spread out along the dorsoventral axis of the crania, and do not touch any bones, which minimized the potential for human error (or interpretation) in our manual segmentations that serve as the ground truth data. The otoliths from the atlas were segmented in the same manner. Using the antsApplyTransforms function of the ANTsR package (Avants, 2020), for each image in our sample we applied the deformable transformation field, generated during the atlas building step, to the manual atlas otolith segmentation. This function essentially maps the atlas otolith segmentation into the subject space of each image by inversing the calculated transformation fields, effectively creating an automated segmentation of the otoliths for each fish. From this mapping, we calculated the volumes of CA derived segmentation and statistically compared them to ground truth manual segmentations using Welch two-sample *t*-tests (Table 1). We also visually inspected all automatically segmented volumes in 3D Slicer to ensure they were segmenting the full and correct structures. All statistical analysis and image registrations were done using the R extensions of the ANTs ecosystem (Avants, 2020).

**Fig. 4.**
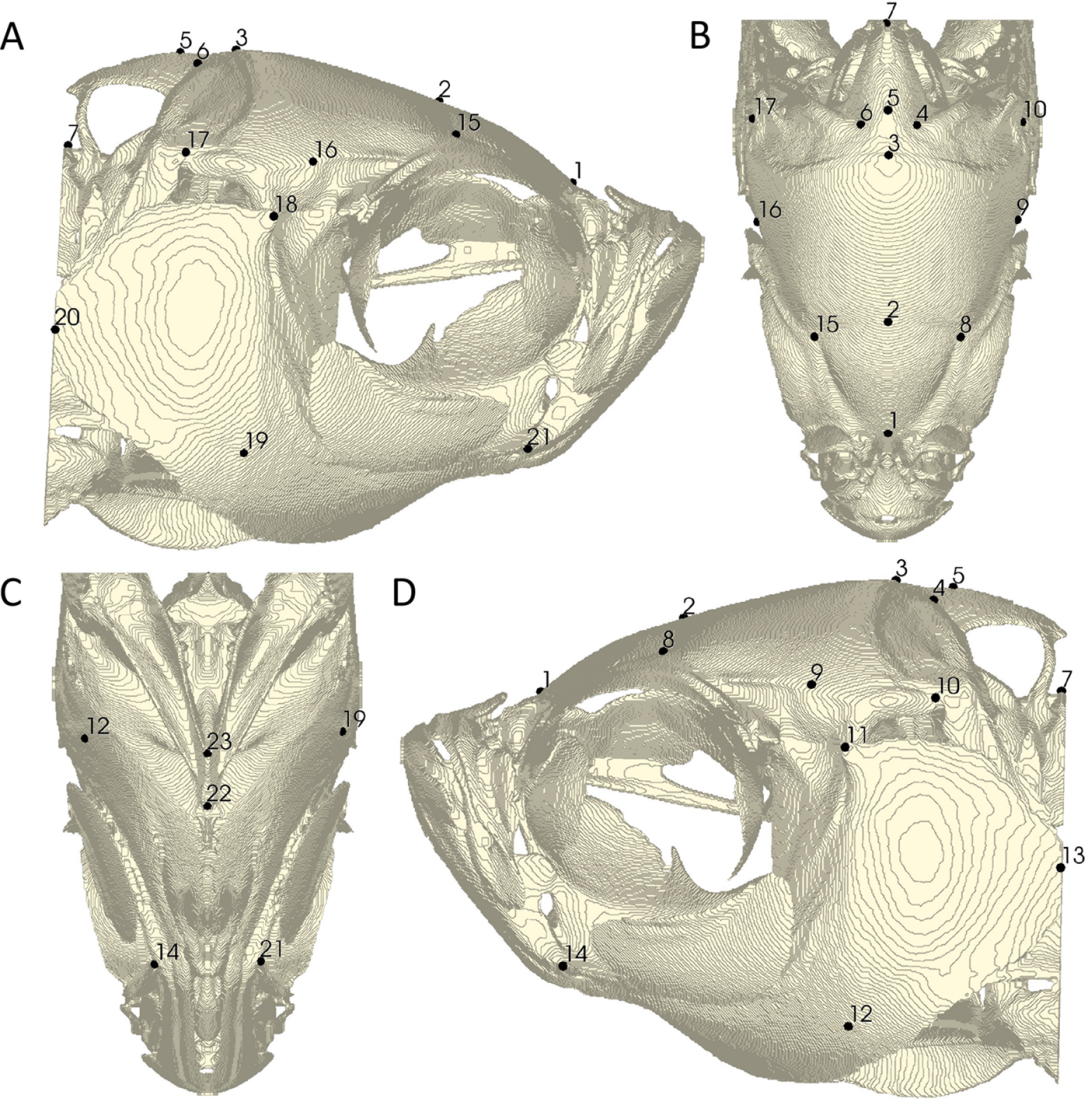
**Gold standard of 23 manual landmarks.** Landmark placement represents the average location of two independent landmark placements by the same author. Right lateral (A), dorsal (B), ventral (C), and left lateral (D) views of the atlas mesh are shown. Landmark definitions can be found in Table 3.

To further validate our atlas and overall pipeline, we placed 23 traditional landmark points on the meshes of each specimen in our sample (Table 3, Fig. 4). Each specimen was landmarked twice by the same author (K.M.D.) and the average of the two were used as the gold standard for comparison. To validate the ALPACA transfer of points, which uses linear and deformable point cloud registration, we used ALPACA to transfer the 23 gold standard points from the atlas to all meshes in the study (Porto et al., 2021) and calculated the Euclidean distance between ALPACA transferred points to the original gold standard landmark points. We also calculated the Euclidean distances between the gold standard landmark points and each of the two manual landmark placements to establish a comparison between the automated and manual landmarking methods and used an ANOVA to test for differences in Euclidean distances from the gold standard among methods overall. Paired Mann–Whitney U test was used to test for differences in digitization errors for each landmark (Table 2).

**Table 3.**
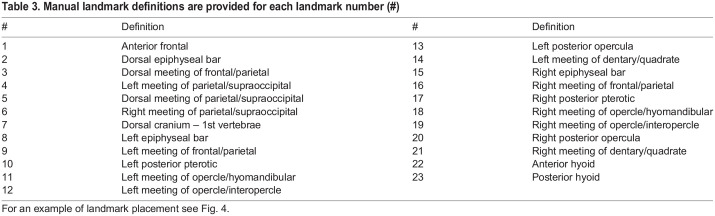
Manual landmark definitions are provided for each landmark number (#)

To compare object sizes and shapes among our methods and groups, we performed a joint generalized Procrustes superimposition on the combined set of gold standard and ALPACA transferred landmark points (Rohlf and Slice, 1990) and ran a Procrustes ANOVA with landmarking method as a factor using the procD.lm function in the geomorph R package (Adams and Otárola-Castillo, 2013). Using the joint superimposition, we also tested whether Procrustes variances of each method are significantly different from each other using a permutation procedure where the vectors of residuals are randomized among methods using the morphol.disparity function in the geomorph R package (Adams and Otárola-Castillo, 2013). Finally, we compared the normalized centroid size, or centroid size divided by the number of landmarks for each method (Toussaint et al., 2021). Centroid size was calculated for each method using the Geomorph package in R (Adams and Otárola-Castillo, 2013).

### Analysis of ZF cranial shape difference in wild types and mutants

**Fig. 5.**
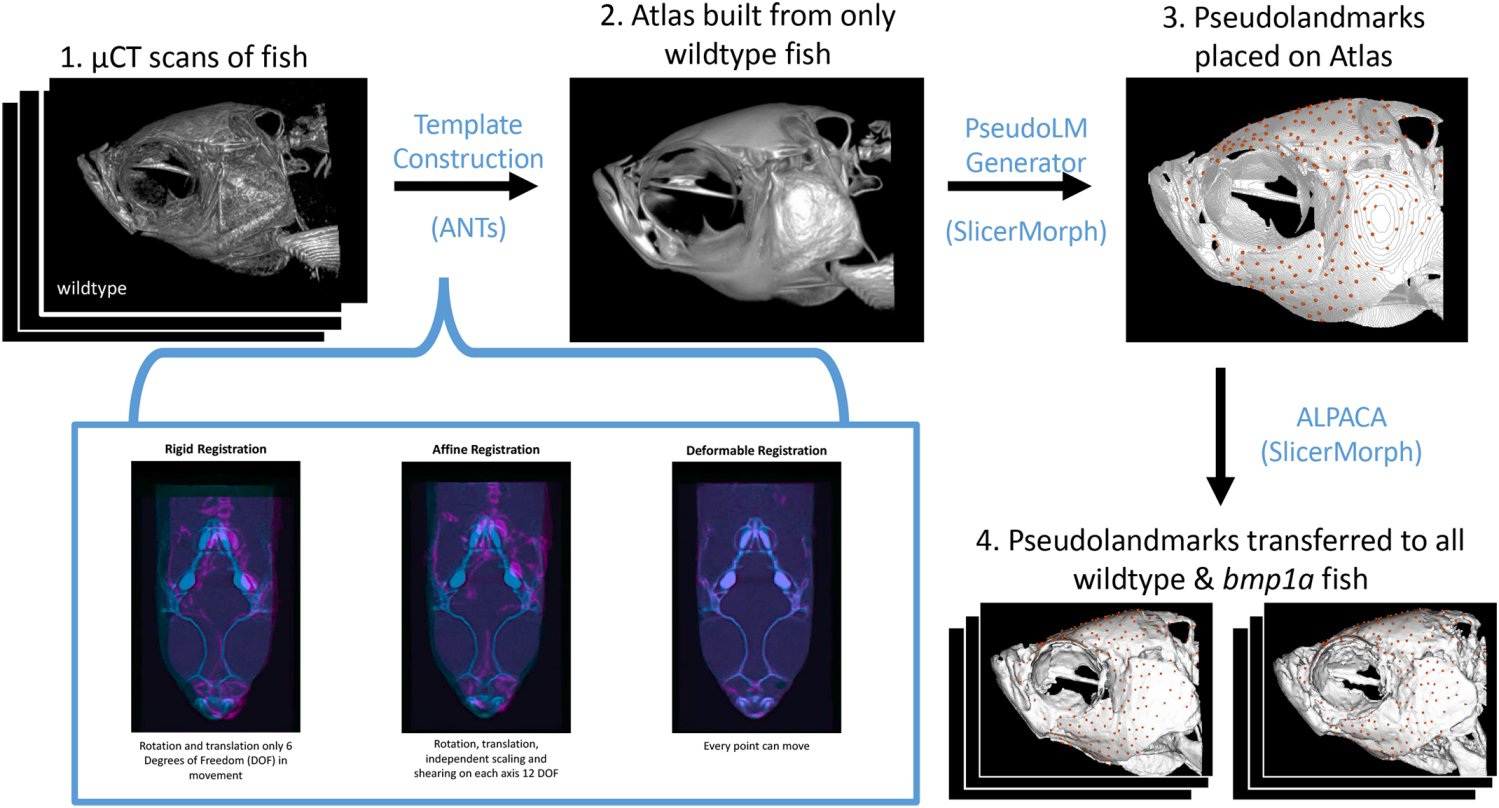
**Pipeline for atlas building, pseudo-landmark generation, and transferring pseudo-landmarks to individual fish.** Blue text notes the software used between each step. (1) Starting with µCT scans of wild-type fish, ANTs uses a series of rigid, affine, and deformable registrations to create an average image, or (2) Atlas. The PseudoLMGenerator tool in SlicerMorph was used to (3) place 372 pseudo-landmarks on the atlas. The ALPACA tool in SlicerMorph was used to (4) transfer points from the atlas to wild-type and bmp1a fish for comparisons between groups.

To identify regions that might differ outside of the manual landmarks, we opted to use a pseudo-landmark-based analysis between *bmp1a* and wild-type fish. To place pseudo-landmark points (pLMs) on each of our specimens, we first created 3D models of from our CT volumes using the Segment Editor module of 3D Slicer (Fedorov et al., 2012). To generate a set of pLMs on our atlas model, we used the PseudoLMGenerator module in the SlicerMorph extension of 3D Slicer which uses the original mesh geometry and a sagittal plane as the axis of symmetry, to generate a dense symmetric set of surface points (Rolfe et al., 2021). One author (K.M.D.) then went through the pLMs and removed points that were on both jaws and the pectoral girdle using the MarkupEditor tool in 3D Slicer (Rolfe et al., 2021; Fig. 5). Both of these structures are highly prone to post-mortem deformation due to handling and preservation, as such they represent confounding non-biological variation and should be excluded from the analysis. The final number of pLMs on the template was 372. To transfer the pLMs from the atlas to all other models in the study, we used the ALPACA module in the SlicerMorph extension of 3D Slicer (Porto et al., 2021). We skipped the optional scaling step of the ALPACA pipeline as all of our samples were of similar size and used default settings (see Porto et al., 2021 for full details on parameter options) to transfer pLMs from the atlas to all meshes in our sample (Fig. 5; Fig. S2).

To examine differences between *bmp1a* and wild-type fish, we ran a Generalized Procrustes Analyses (GPA) on the all pLMs, allowing all pLMs to slide along the surface using the gpagen function of the geomorph (Adams and Otárola-Castillo, 2013). We ran a symmetry analysis on the GPA coordinates using the bilat.symmetry function in geomorph (Adams and Otárola-Castillo, 2013). From this output, we first ran Procrustes ANOVAs using the procD.lm function in geomorph to determine if the symmetric and fluctuating asymmetric components of shape variation differ between groups. We also ran separate principal components analyses on both the symmetric and asymmetric components of variation from the symmetry analysis using the geomorph (Adams and Otárola-Castillo, 2013). Visualizations were created in the SlicerMorph extension of 3D Slicer (Rolfe et al., 2021) and using ggplot in R (Wickham, 2016).

## Supplementary Material

Supplementary information
