# Supplementary material for: Computational anatomy and geometric shape analysis enables analysis of complex craniofacial phenotypes in zebrafish
Source: Biol Open. 2022 Feb 17;11(2):bio058948. doi: 10.1242/bio.058948 (PMC8864294; doi:10.1242/bio.058948)
Supplement: Supplementary information [file biolopen-11-058948-s1.pdf]

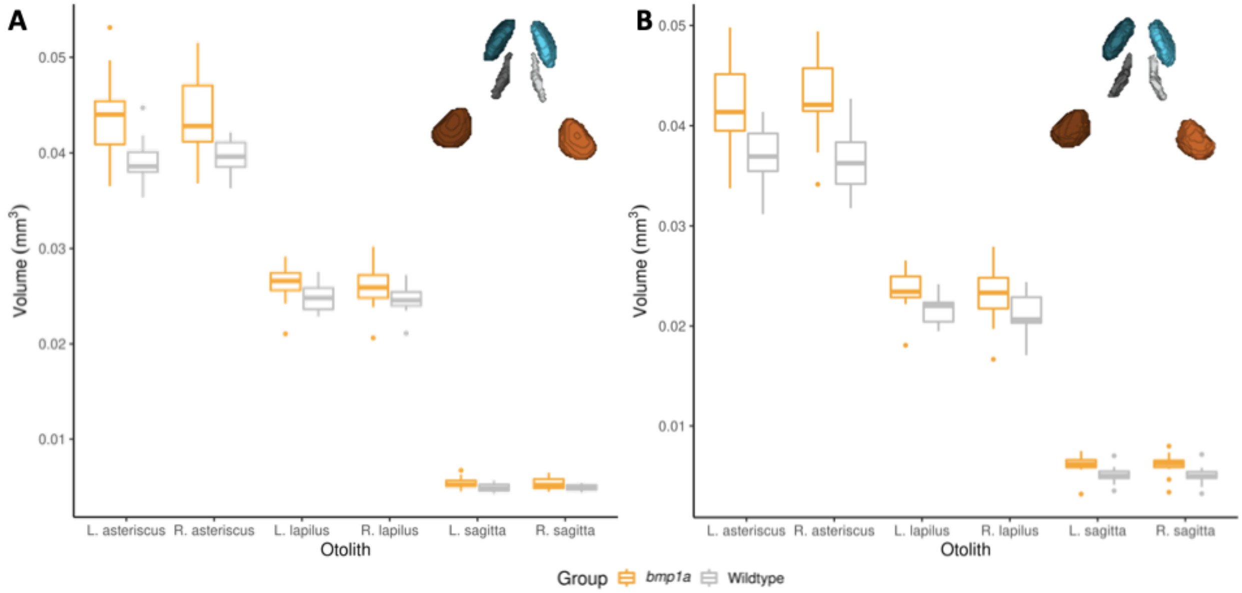

Fig. S1. Boxplots of segmented otolith volumes. Wildtype (grey) and *bmp1a* mutant (orange) boxplots are shown for each of the six otoliths for both the (A) manually segmented volumes and (B) atlas segmented volumes. For each otolith, mutants have larger median volumes than wildtype fish (Table 1). Insets show the dorsal view of otolith segments (asterisk in blue, lapilus in orange, and sagitta in grey) with lighter colors on the left side of the head, and darker colors on the right.

Biology Open • Supplementary information

Biology Open (2022): doi:10.1242/bio.058948: Supplementary information

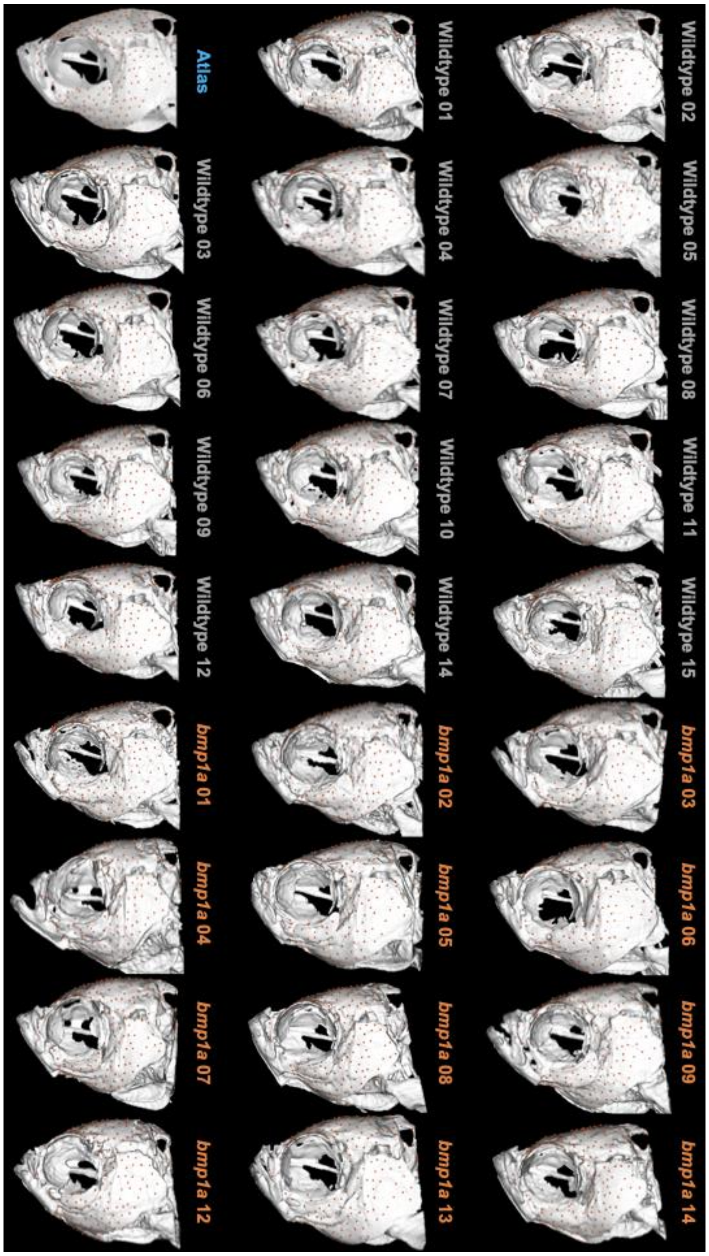

Fig. S2. Left lateral view of fish meshes with pseudo-landmarks generated from the PseudoLMGenerator and transverse using the ALPACA modules in SlicerMorph. Name color represents groupings, the atlas is in blue, wildtype fish are in grey and *bmp1a* fish are in orange.

Biology Open • Supplementary information

Table S1. Welch two sample t-test for difference between manual and atlas-segmented otolith volumes for *bmp1a* and wildtype fish. We provide the mean volumes(x) for each method, degrees of freedom (df), test statistic (t), p value (p), and confidence interval (UCL-LCL).

Otolith	group	x atlas (mm^3)	x manual (mm^3)	df	t	p	UCL	LCL
Left asteriscus	*bmp1a*	0.042	0.044	21.971	-0.902	0.377	-0.006	0.044
Left asteriscus	wildtype	0.037	0.039	25.117	-1.998	0.057	-3.995	6.056
Right asteriscus	*bmp1a*	0.043	0.044	21.997	-0.618	0.543	-0.005	0.003
Right asteriscus	wildtype	0.037	0.040	20.891	-2.744	0.012	-0.005	-0.0007
Left lapilus	*bmp1a*	0.024	0.026	22.000	-3.074	0.006	-0.005	-0.0009
Left lapilus	wildtype	0.022	0.025	25.581	-5.438	<0.001	-0.004	-0.002
Right lapilus	*bmp1a*	0.023	0.026	20.94	-2.490	0.021	-0.005	-0.0005
Right lapilus	wildtype	0.021	0.025	24.294	-5.347	<0.001	-0.005	-0.002
Left sagitta	*bmp1a*	0.006	0.005	17.619	1.998	0.061	-0.00004	-0.002
Left sagitta	wildtype	0.005	0.005	19.564	0.799	0.434	-0.0003	0.0007
Right sagitta	*bmp1a*	0.006	0.005	16.603	1.934	0.070	-0.0006	0.002
Right sagitta	wildtype	0.005	0.005	15.959	0.236	0.816	-0.0005	0.0005

Biology Open • Supplementary information

Table S2. Principle component (PC) scores for first 10 PCs of symmetric component of shape analysis.

	PC1	PC2	PC3	PC4	PC5	PC6	PC7	PC8	PC9	PC10
symm_sham_atlas	-0.0045	-0.0071	-0.0076	-0.0019	0.0071	0.0025	0.0029	-0.0026	-0.0052	-0.0009
msbl95ABsham15	0.0195	-0.0157	0.0059	-0.0056	0.0007	-0.0080	-0.0185	-0.0061	-0.0050	0.0024
msbl95ABsham14	-0.0326	0.0036	0.0023	0.0043	0.0063	-0.0040	-0.0015	-0.0022	0.0091	-0.0039
msbl95ABsham12	-0.0424	0.0012	0.0210	0.0118	-0.0092	0.0058	-0.0007	-0.0028	0.0014	-0.0011
msbl95ABsham11	-0.0202	-0.0220	-0.0059	0.0103	-0.0073	0.0002	0.0013	-0.0054	-0.0084	0.0038
msbl95ABsham10	0.0214	-0.0182	0.0026	-0.0023	0.0067	0.0072	-0.0030	0.0052	-0.0040	-0.0028
msbl95ABsham09	0.0316	-0.0092	0.0030	-0.0064	0.0001	0.0040	0.0039	0.0089	0.0026	-0.0072
msbl95ABsham08	-0.0032	-0.0131	0.0013	-0.0078	-0.0123	0.0008	0.0033	-0.0034	-0.0033	-0.0038
msbl95ABsham07	-0.0006	-0.0147	-0.0180	-0.0021	0.0062	-0.0041	-0.0055	0.0024	0.0102	0.0000
msbl95ABsham06	-0.0358	-0.0054	-0.0175	0.0041	0.0044	0.0038	0.0012	0.0036	0.0048	0.0011
msbl95ABsham05	-0.0354	0.0110	0.0002	-0.0092	-0.0015	0.0100	-0.0071	0.0002	-0.0044	-0.0095
msbl95ABsham04	-0.0390	0.0025	-0.0056	0.0004	-0.0020	-0.0073	0.0047	0.0025	-0.0047	0.0042
msbl95ABsham03	0.0036	0.0029	-0.0089	-0.0222	0.0019	0.0024	0.0031	0.0022	-0.0083	0.0069
msbl95ABsham02	0.0101	-0.0146	0.0059	-0.0007	0.0086	-0.0070	0.0028	0.0005	0.0028	-0.0043
msbl95ABsham01	-0.0055	-0.0039	0.0039	-0.0039	-0.0143	-0.0037	0.0017	-0.0059	0.0022	-0.0010
msbl_95AB_bmp1a_crispant_14	0.0145	-0.0023	0.0312	0.0153	0.0128	0.0001	0.0070	0.0037	-0.0057	0.0013
msbl_95AB_bmp1a_crispant_13	0.0221	0.0113	-0.0032	-0.0025	-0.0030	0.0054	-0.0009	-0.0075	0.0022	-0.0093
msbl_95AB_bmp1a_crispant_12	0.0149	0.0048	-0.0126	0.0067	-0.0053	0.0253	0.0016	0.0048	0.0013	0.0056
msbl_95AB_bmp1a_crispant_09	0.0043	0.0178	-0.0079	-0.0102	-0.0017	-0.0154	0.0081	0.0065	-0.0055	-0.0064
msbl_95AB_bmp1a_crispant_08	-0.0028	0.0064	-0.0014	0.0140	-0.0136	-0.0106	0.0037	0.0074	0.0022	0.0026
msbl_95AB_bmp1a_crispant_07	0.0118	0.0189	-0.0022	0.0147	0.0108	0.0045	0.0054	-0.0095	-0.0012	-0.0015
msbl_95AB_bmp1a_crispant_06	0.0178	-0.0209	-0.0036	0.0099	-0.0003	-0.0034	0.0036	-0.0001	0.0059	0.0024
msbl_95AB_bmp1a_crispant_05	-0.0034	0.0112	0.0164	-0.0070	0.0023	0.0017	-0.0078	0.0067	0.0015	0.0056
msbl_95AB_bmp1a_crispant_04	0.0505	0.0205	-0.0161	0.0203	-0.0040	-0.0064	-0.0077	-0.0018	-0.0037	0.0010
msbl_95AB_bmp1a_crispant_03	0.0265	0.0063	0.0088	-0.0212	-0.0012	0.0005	0.0102	-0.0104	0.0079	0.0076
msbl_95AB_bmp1a_crispant_02	0.0083	0.0148	0.0129	-0.0054	-0.0086	-0.0006	-0.0083	0.0056	0.0051	0.0028
msbl_95AB_bmp1a_crispant_01	-0.0317	0.0142	-0.0048	-0.0034	0.0166	-0.0039	-0.0037	-0.0026	0.0000	0.0042

Table S3. Principle component (PC) scores for first 10 PCs of asymmetric component of shape analysis.

	PC1	PC2	PC3	PC4	PC5	PC6	PC7	PC8	PC9	PC10
symm_sham_atlas	-0.0045	-0.0071	-0.0076	-0.0019	0.0071	0.0025	0.0029	-0.0026	-0.0052	-0.0009
msbl95ABsham15	0.0195	-0.0157	0.0059	-0.0056	0.0007	-0.0080	-0.0185	-0.0061	-0.0050	0.0024
msbl95ABsham14	-0.0326	0.0036	0.0023	0.0043	0.0063	-0.0040	-0.0015	-0.0022	0.0091	-0.0039
msbl95ABsham12	-0.0424	0.0012	0.0210	0.0118	-0.0092	0.0058	-0.0007	-0.0028	0.0014	-0.0011
msbl95ABsham11	-0.0202	-0.0220	-0.0059	0.0103	-0.0073	0.0002	0.0013	-0.0054	-0.0084	0.0038
msbl95ABsham10	0.0214	-0.0182	0.0026	-0.0023	0.0067	0.0072	-0.0030	0.0052	-0.0040	-0.0028
msbl95ABsham09	0.0316	-0.0092	0.0030	-0.0064	0.0001	0.0040	0.0039	0.0089	0.0026	-0.0072
msbl95ABsham08	-0.0032	-0.0131	0.0013	-0.0078	-0.0123	0.0008	0.0033	-0.0034	-0.0033	-0.0038
msbl95ABsham07	-0.0006	-0.0147	-0.0180	-0.0021	0.0062	-0.0041	-0.0055	0.0024	0.0102	0.0000
msbl95ABsham06	-0.0358	-0.0054	-0.0175	0.0041	0.0044	0.0038	0.0012	0.0036	0.0048	0.0011
msbl95ABsham05	-0.0354	0.0110	0.0002	-0.0092	-0.0015	0.0100	-0.0071	0.0002	-0.0044	-0.0095
msbl95ABsham04	-0.0390	0.0025	-0.0056	0.0004	-0.0020	-0.0073	0.0047	0.0025	-0.0047	0.0042
msbl95ABsham03	0.0036	0.0029	-0.0089	-0.0222	0.0019	0.0024	0.0031	0.0022	-0.0083	0.0069
msbl95ABsham02	0.0101	-0.0146	0.0059	-0.0007	0.0086	-0.0070	0.0028	0.0005	0.0028	-0.0043
msbl95ABsham01	-0.0055	-0.0039	0.0039	-0.0039	-0.0143	-0.0037	0.0017	-0.0059	0.0022	-0.0010
msbl_95AB_bmp1a_crispant_14	0.0145	-0.0023	0.0312	0.0153	0.0128	0.0001	0.0070	0.0037	-0.0057	0.0013
msbl_95AB_bmp1a_crispant_13	0.0221	0.0113	-0.0032	-0.0025	-0.0030	0.0054	-0.0009	-0.0075	0.0022	-0.0093
msbl_95AB_bmp1a_crispant_12	0.0149	0.0048	-0.0126	0.0067	-0.0053	0.0253	0.0016	0.0048	0.0013	0.0056
msbl_95AB_bmp1a_crispant_09	0.0043	0.0178	-0.0079	-0.0102	-0.0017	-0.0154	0.0081	0.0065	-0.0055	-0.0064
msbl_95AB_bmp1a_crispant_08	-0.0028	0.0064	-0.0014	0.0140	-0.0136	-0.0106	0.0037	0.0074	0.0022	0.0026
msbl_95AB_bmp1a_crispant_07	0.0118	0.0189	-0.0022	0.0147	0.0108	0.0045	0.0054	-0.0095	-0.0012	-0.0015
msbl_95AB_bmp1a_crispant_06	0.0178	-0.0209	-0.0036	0.0099	-0.0003	-0.0034	0.0036	-0.0001	0.0059	0.0024
msbl_95AB_bmp1a_crispant_05	-0.0034	0.0112	0.0164	-0.0070	0.0023	0.0017	-0.0078	0.0067	0.0015	0.0056
msbl_95AB_bmp1a_crispant_04	0.0505	0.0205	-0.0161	0.0203	-0.0040	-0.0064	-0.0077	-0.0018	-0.0037	0.0010
msbl_95AB_bmp1a_crispant_03	0.0265	0.0063	0.0088	-0.0212	-0.0012	0.0005	0.0102	-0.0104	0.0079	0.0076
msbl_95AB_bmp1a_crispant_02	0.0083	0.0148	0.0129	-0.0054	-0.0086	-0.0006	-0.0083	0.0056	0.0051	0.0028
msbl_95AB_bmp1a_crispant_01	-0.0317	0.0142	-0.0048	-0.0034	0.0166	-0.0039	-0.0037	-0.0026	0.0000	0.0042
